# Management of pneumothorax with 8.3-French Pigtail Catheter: description of the ultrasound-guided technique and case series

**DOI:** 10.1186/s13089-022-00303-4

**Published:** 2023-01-12

**Authors:** Camilo Ramírez-Giraldo, Carlos Eduardo Rey-Chaves, David Rene Rodriguez Lima

**Affiliations:** 1 Department of Surgery, Hospital Universitario Mayor – Méderi, Bogotá, Colombia; 2 Department of Surgery, Universidad del Rosario, Bogotá, Colombia; 3 Grupo de Investigación Clínica, Escuela de Medicina y Ciencias de La Salud, Universidad del Rosario, Bogotá, Colombia; 4 Critical and Intensive Care Medicine, Hospital Universitario Mayor - Méderi, Bogotá, Colombia

**Keywords:** Pneumothorax, Pigtail, Thoracostomy, Chest tube, Ultrasound

## Abstract

Spontaneous and traumatic pneumothorax are most often treated with chest tube (CT) thoracostomy. However, it appears that small-bore drainage systems have similar success rates with lower complications, pain, and discomfort for the patient. We present the description of the ultrasound-guided technique for pneumothorax drainage with an 8.3-French pigtail catheter (PC) in a case series of 10 patients.

## Background

Pneumothorax is defined as the presence of air in the pleural cavity; it can be secondary to underlying pulmonary pathology or trauma. In trauma, approximately 40–50% of thoracic injuries develop pneumothorax [1]. Traditionally, following the suggestion of the American College of Chest Physicians and the Advanced Trauma Life Support (ATLS), all traumatic pneumothoraxes must be treated with a pleural drainage that could be chest tube (CT) or pigtail catheter (PC) [1, 2]; nonetheless, there are no consensus on the size and location of the drainage, and it is still matter of concern since CT thoracostomy is a painful invasive procedure that increases hospital stay and other complications [2, 3].

For this reason, the use of techniques with small-bore PC instead of a CT is increasing. Chang et al. in a meta-analysis demonstrated that the use of PC in the treatment of pneumothorax could replace the use of CT because it shows lesser rates of complications and reduces hospital stay and recurrences [4].

Following the improvement in the management of patients with traumatic pneumothorax, a recent clinical trial compared the use of 14-French PC versus 29–32-French CT, with similar baseline and clinical characteristics, such as comparable outcomes between both approaches, in terms of complication and success rate; however, PC shows a better perception experience of the patient [5].

This article aims to describe the ultrasound-guided technique for pneumothorax drainage with an 8.3-French PC used in a highly complex hospital and their initial clinical results.

## Methods

### Study population

With the Institutional Review Board’s approval and following Health Insurance Portability and Accountability Act (HIPAA) guidelines, a retrospective review of a prospectively collected database was conducted. All patients over 18 years of age who underwent pneumothorax drainage using pigtail catheter between January 2021 and December 2021 were included. Patients with no surgical description and missing data were excluded.

The percentage pneumothorax size was quantified as a linear function of the interpleural distance (ID), where ID is calculated as the sum of three distances: the maximum apical interpleural distance (A), the interpleural distance at the midpoint of the upper half of the lung (B), and the interpleural distance at the midpoint of the lower half of the lung (C). This method described by Collins [6] uses the following equation:

$$\% {\text{ pneumothorax}} = { 4}.{2 } + { 4}.{7 }\left( {{\text{A }} + {\text{ B }} + {\text{ C}}} \right) \, = { 4}.{2 } + { 4}.{7 }\left( {{\text{ID}}} \right).$$

Ethical compliance with the Helsinki Declaration, current legislation on research Res. 008430-1993 and Res. 2378-2008 (Colombia), and the International Committee of Medical Journal Editors (ICMJE) were ensured under our Ethics and Research Institutional Committee (IRB) approval. Upon admission to the institution, patients gave a written informed consent to use their clinical information for research purposes.

### Statistical analysis

Demographic, clinical, surgical, and outcome variables were described. Categorical variables were described as proportions and continuous variables as medians with their respective interquartile range (IQR).

### Technique description

**Fig. 1:**
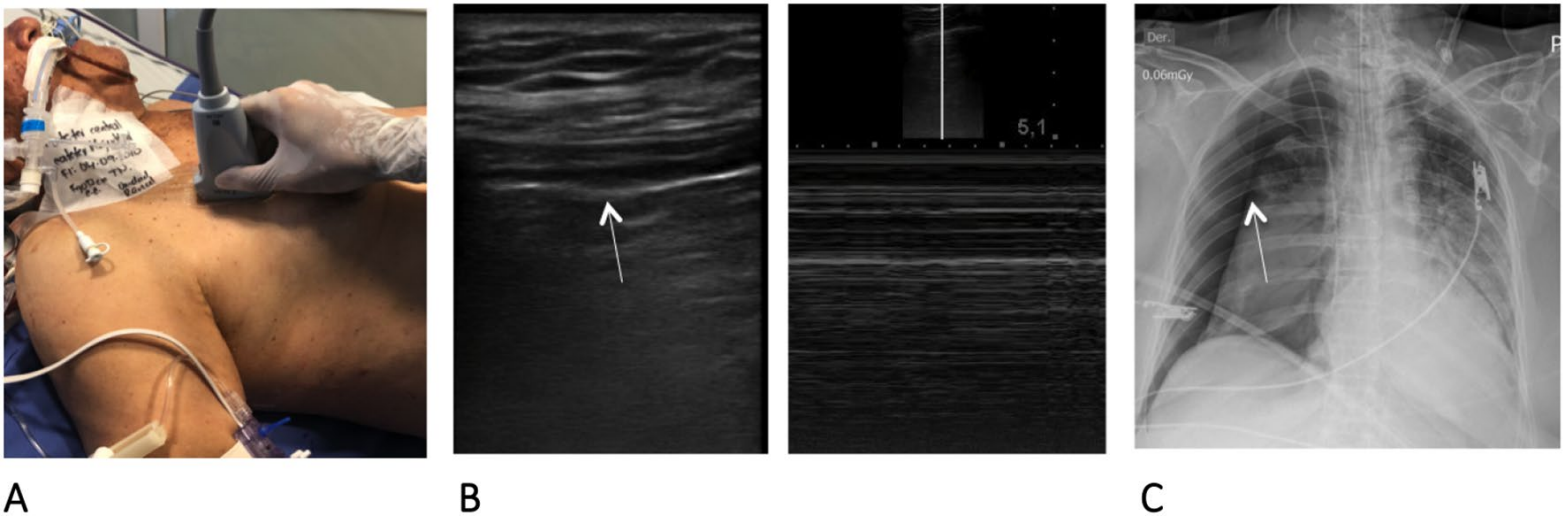
**A** Evaluation of the presence of pneumothorax with a high-frequency linear transducer (13 MHz) **B.** Pneumothorax was diagnosed due to the absence of pleural sliding (arrow) and the barcode sign in M-mode **C.** Chest radiograph confirming the presence of pneumothorax (arrow)

**Fig. 2:**
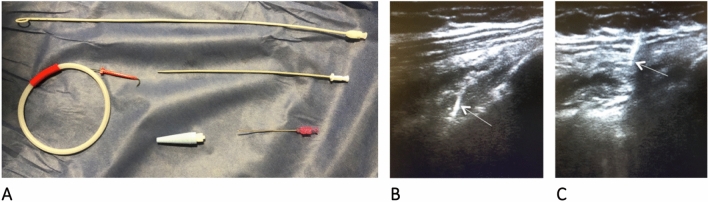
**A** Thoracentesis kit **B.** Evaluation with a 13-MHz linear transducer at the level of the fifth to seventh intercostal space along the mid-axillary line, verifying the absence of pleural sliding at the puncture site; once vessel interposition is ruled out with color Doppler, the puncture is made; the passage of the needle to the pleural space is visualized in real time; visualization is lost upon entry into the pleural space given the presence of air (arrow) **C.** A guide wire is passed, verifying entry into the pleural space (arrow); as with the needle, visualization is lost when entering the pleural space

**Fig. 3:**
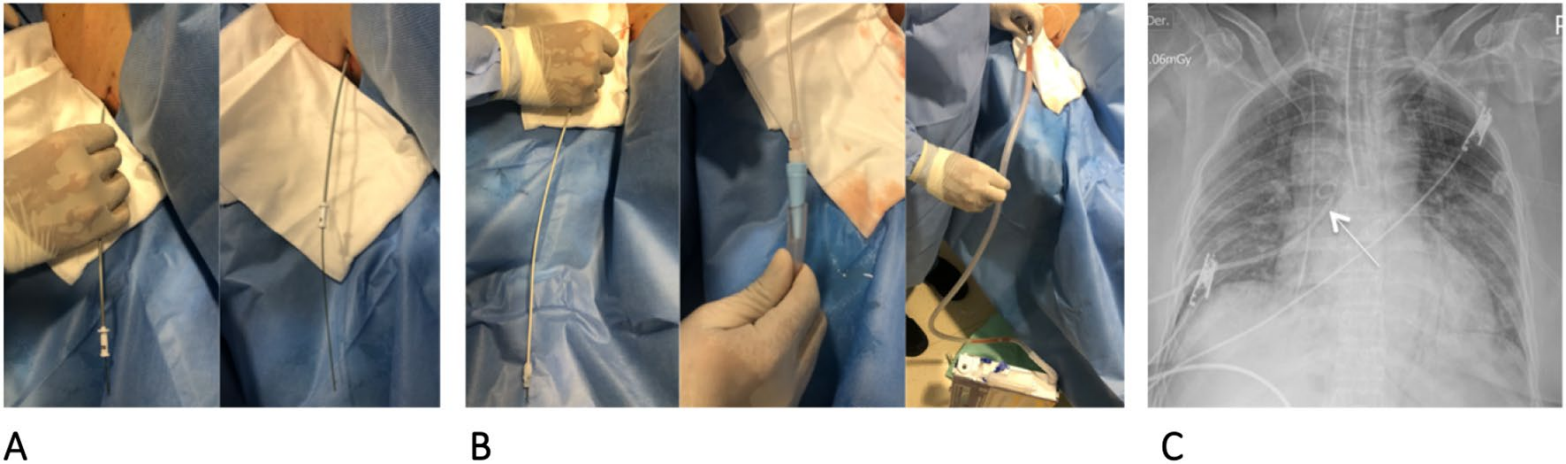
**A** Passage of the dilator, between 1 and 2 cm, depending on the thickness of the chest wall **B.** Passage of the pigtail catheter and connection to a thoracic drainage system **C.** Chest X-ray with complete lung re-expansion and pigtail in position (arrow)

For the procedure, a Sonosite M-Turbo ultrasound machine with a 13-MHz linear transducer is used. The recommended technique is described as follows (Figs. 1, 2, and 3):

1. The thoracentesis kit, which contains antiseptic solution, sterile gloves, sterile gauze, subcutaneous needle, and 10-ml syringe to administer local anesthetics, 16-gauge needle, J-tip guide wire, 8-French (Fr) dilator, 40-cm × 8.3-Fr pigtail catheter, chest drainage system, and catheter connector to this system, is prepared.
2. The patient is placed in a supine position with 45° head elevation.
3. The pneumothorax is visualized by ultrasound with a 13-MHz high-frequency linear transducer, confirmed by the absence of pleural sliding, presence of the barcode sign, and, if possible, location of the lung point.
4. Although ultrasound diagnosis is very sensitive, in the absence of suspicion of obstructive shock due to tension pneumothorax, we recommend taking a chest X-ray, given that depending on the clinical situation and the size of the pneumothorax, conservative management with radiological monitoring can sometimes be indicated.
5. Once it is indicated that the pneumothorax requires drainage, after asepsis and antisepsis, the linear transducer is positioned on the mid-axillary line between the fifth and seventh intercostal space, always making sure that it is above the diaphragm.
6. With color Doppler, no intercostal vessel interposition is observed at the site selected for puncture.
7. The 16-gauge needle is inserted into the selected space under direct view (the selected space must have loss of pleural sliding and the barcode sign); once the pleural space is entered and air has entered, visualization of the needle tip is lost; therefore, the needle should not be inserted beyond an additional 1 cm.
8. The J-tip guide wire is then advanced; it is introduced approximately 15 to 20 cm, and its entry into the pleural space is verified; once again, its position in the pleural space cannot be verified due to the presence of air.
9. The 8.0-Fr dilator is passed 1 to 2 cm, depending on the thickness of the chest wall.
10. The dilator is removed, and the 8.3-Fr pigtail catheter is inserted, advanced 15 to 20 cm, and connected to a chest drainage system.
11. The catheter remains connected to a -20 cmH_2_O suction system, and the presence of pleural sliding is verified with ultrasound. If it is present, the lung is re-expanded. However, visualization is not always possible; therefore, in cases in which pleural sliding is not re-established, a control chest radiograph should be performed.

## Results

A total of 10 patients underwent ultrasound-guided technique for pneumothorax drainage with an 8.3-French pigtail catheter (PC). 70% of patients were male. The median age was 65 (IQR: 40.5–71.7) years. The median BMI was 28.7 (IQR:23.8–29.6) Kg/m^2^. In most of the patients (60%) pneumothorax was secondary to traumatic injuries. Coronavirus infection was present in two patients (20%).

The median volume of pneumothorax calculated by chest radiographic was 20.5% (IQR:19.25–37.5); only 2 patients present with pleural effusion associated. The median duration of the drainage was 2.5 (IQR:2.0–4.2) days. The mean of in-hospital stay was 3 (IQR: 3.0–4.5) days. No complications were observed after 30 days of follow-up, with a 0% of mortality rate. All cases were successfully treated, and with no recurrence after the follow-up.

## Discussion

Conventionally, thoracic impairments, such as pneumothorax (primary or secondary) and hemothorax, are managed with large CT placement independent of the size [1]; and historically, there is a belief that large-bore chest tubes show an increased rate of success in the management of these pathologies, even in pleural effusions [1, 4].

However, the present-day literature focuses on the less invasive techniques in the management of traumatic or spontaneous pneumothorax [7–10]. Kulvatunyou et al. [11], in a randomized clinical trial, showed that there is no difference in clinical outcomes comparing PC versus CT in patients with traumatic pneumothorax, with benefit in pain relief in patients with PC, with the lesser failure rate. [10]. Other studies evaluated the impact of the large/small CT in the management of hemo/pneumothorax; however, according to Inaba et al. [12], there are no differences in clinical outcomes, such as efficacy, rate of complications, and needs for additional procedures. All these data impact the clinical guidelines of ATLS, which recommends small CT placement for all cases of pneumothorax [1, 2].

The use of less invasive techniques is increasing, such as the utilization of PC in the management of pneumothorax. Multiple studies [7–10] are now evaluating the advantages and benefits of this approach; Bauman et al. [5] show that in terms of failure rates there is no difference between CT and PC, with a better patient’s experience, comparable results with Kulvatunyou et al. [11].

Chang et al. [4] in a meta-analysis concluded that placement of PC for traumatic or non-traumatic pneumothorax shows less rate of complications, shorter duration of the drainage and hospital stay with statistically significant differences, compared with large CT, these results could impact in the clinical outcomes of the patients and financial burden. According to Fang et al. [8] meta-analysis increases the evidence in favor of the advantages of PC in the management of pneumothorax independent of the cause or size, showing a success rate between 72 and 88% and complication rates between 9 and 18%.

However, other authors include PC in the management in other pathologies, such as pleural effusions, Rodriguez Lima et al. [13] with a prospective study including patients critically ill, with associated pleural effusions, showing a 1.2% complications rate, lesser than the reported in the literature, with comparable rates of success.

Tsai et al. [14] in a retrospective study reported that treatment failure rates were 42.9%, 25.9%, and 15.5% in patients with pneumothorax sizes > 62.6%, 38–62 0.6%, and < 38%. The median pneumothorax size in this case series was 20.5%, which may partly explain our 100% success rate. However, in different observational studies the diameter of the drain does not seem to be the most important factor for successful re-expansion [15–17]. As in the technique described by us, we found an adequate success rate despite being one of the smallest diameters reported in the literature. In addition, it seems more important for the success of the re-expansion that the tip of the drain is located at the apex of the pleural cavity, regardless of the type of drain inserted [18].

Benton et al. [18] in a descriptive study demonstrated displacement was higher with small bore (21% vs 8%); however, in our series we did not observe any unplanned removal. All PCs were fixed to the skin with silk 2–0.

Our paper describes the operative technique with real-time ultrasound guided of #8.3 French PC in the management of pneumothorax independent of the cause and the size. Our initial results show a success rate slightly higher than the one described in the literature (82% vs 100%). Some series of cases report a complication rate between 1.2% and 9%; in our series of cases, no complications secondary to the procedure were evidenced. Duration of the drainage was similar to those reported in Chang et al. [4] with a mean of 3 days ± 1.41 days. Also, no recurrence rates were defined, and any patient required CT placement or invasive procedures after the PC with real-time ultrasound approach.

To the best of our knowledge, this is the first study that describes the operative technique of PC with real-time ultrasound-guided placement for the management of pneumothorax with small-size PC. These initial results show promising outcomes, with a low rate of complications and morbidity, as well, a high rate of success, with shorter in-hospital stay and duration of the drainage.

Limitations of our study include the retrospective nature and the small number of patients. However, this paper describes the operative technique with real-time ultrasound that shows the advantages of the use of less invasive techniques in traumatic or spontaneous pneumothorax.

## Conclusion

The standardized operative technique with real-time ultrasound of PC placement in traumatic or spontaneous pneumothorax shows promising outcomes, in terms of rates of success, morbidity, and mortality, and could be effective as a single treatment. However, further prospective and randomized trials need to be performed.

## Data Availability

All data used during this study are available by email at the request of the editorial committee.

## Abbreviations

ATLS: Advanced Trauma Life Support
CT: Chest tube
PC: Pigtail catheter
ID: Interpleural distance

## References

[1] Tran J, Haussner W, Shah K (2021). Traumatic pneumothorax: a review of current diagnostic practices and evolving management. J Emerg Med.

[2] Galvagno SM, Nahmias JT, Young DA (2019). Advanced trauma life support^®^ update 2019: management and applications for adults and special populations. Anesthesiol Clin.

[3] Munnell ER (1997). Thoracic drainage. Ann Thorac Surg.

[4] Chang SH, Kang YN, Chiu HY, Chiu YH (2018). A systematic review and meta-analysis comparing pigtail catheter and chest tube as the initial treatment for pneumothorax. Chest.

[5] Bauman ZM, Kulvatunyou N, Joseph B, Gries L, O’Keeffe T, Tang AL (2021). Randomized clinical trial of 14-French (14F) pigtail catheters versus 28–32F chest tubes in the management of patients with traumatic hemothorax and hemopneumothorax. World J Surg.

[6] Collins CD, Lopez A, Mathie A, Wood V, Jackson JE, Roddie ME. Quantification of pneumothorax size on chest radiographs using interpleural distances: regression analysis based on volume measurements from helical CT. AJR Am J Roentgenol. 1995 Nov;165(5):1127-30.10.2214/ajr.165.5.7572489. PMID: 7572489. 10.2214/ajr.165.5.7572489 7572489

[7] Maezawa T, Yanai M, Huh JY, Ariyoshi K (2020). Effectiveness and safety of small-bore tube thoracostomy (≤ 20 Fr) for chest trauma patients : a retrospective observational study. Am J Emerg Med.

[8] Fang M, Liu G, Luo G, Wu T (2018). Does pigtail catheters relieve pneumothorax?. Medicine (Baltimore).

[9] Aziz F, Penupolu S, Flores D (2012). Efficacy of percutaneous pigtail catheters for thoracostomy at bedside. J Thorac Dis.

[10] Lin Y, Tu C, Liang S, Chen H, Chen W, Hsia T (2010). Pigtail catheter for the management of pneumothorax in mechanically ventilated patients. Am J Emerg Med.

[11] Kulvatunyou N, Erickson L, Vijayasekaran A, Gries L, Joseph B, Friese RF (2014). Randomized clinical trial of pigtail catheter versus chest tube in injured patients with uncomplicated traumatic pneumothorax. Br J Surg.

[12] Inaba K, Lustenberger T, Recinos G, Georgiou C, Velmahos GC, Brown C (2010). Does size matter? A prospective analysis of 28–32 versus 36–40 French chest tube size in trauma. J Trauma Acute Care Surg.

[13] Rodriguez Lima DR, Yepes AF, Birchenall Jiménez CI, Mercado Díaz MA, Pinilla Rojas DI (2020). Real-time ultrasound-guided thoracentesis in the intensive care unit: prevalence of mechanical complications. Ultrasound J.

[14] Tsai T, Lin M, Li Y, Chang C, Liao H, Hsu H (2017). The size of spontaneous pneumothorax is a predictor of unsuccessful catheter drainage. Sci Rep.

[15] Takeda S, Nagata N, Yoshida Y, Matsumoto T, Aoyama T, Harada T (2018). Study of the usefulness of small-bore aspiration catheters (Aspiration Kit®) for treating pneumothorax. Respir Investig.

[16] Liman ST, Elicora A, Akgul AG, Topcu S, Özbay S, Mehmetoǧlu SS (2014). Is a small-bore catheter efficient for most pleural pathologies?. Surg Today.

[17] Tsai WK, Chen W, Chih LJ, Cheng WE, Chen CH, Hsu WH (2006). Pigtail catheters vs large-bore chest tubes for management of secondary spontaneous pneumothoraces in adults. Am J Emerg Med.

[18] Benton IJ, Benfield GFA (2009). Comparison of a large and small-calibre tube drain for managing spontaneous pneumothoraces. Respir Med.

